# Jinhong decoction protects sepsis-associated acute lung injury by reducing intestinal bacterial translocation and improving gut microbial homeostasis

**DOI:** 10.3389/fphar.2023.1079482

**Published:** 2023-04-04

**Authors:** Kaifan Bao, Meiling Wang, Li Liu, Dongya Zhang, Cuiyuan Jin, Junfeng Zhang, Liyun Shi

**Affiliations:** ^1^ Department of Immunology, School of Medicine and Holistic Integrative Medicine, Nanjing University of Chinese Medicine, Nanjing, Jiangsu, China; ^2^ Department of Medical Microbiology, School of Medicine and Holistic Integrative Medicine, Nanjing University of Chinese Medicine, Nanjing, Jiangsu, China; ^3^ Institute of Translational Medicine, Zhejiang Shuren University, Hangzhou, Zhejiang, China

**Keywords:** JHD, sepsis, gut microbiome, bacterial translocation, acute lung injury

## Abstract

**Background:** Currently no specific treatments are available for sepsis and the associated syndromes including acute lung injury (ALI). Jinhong Decoction (JHD) is a traditional Chinese prescription, and it has been applied clinically as an efficient and safe treatment for sepsis, but the underlying mechanism remains unknown. The aim of the study was to explore the potential mechanisms of JHD ameliorating sepsis and concurrent ALI.

**Methods:** The cecum ligation puncture (CLP)- induced murine sepsis model was established for determining the efficacy of JHD protecting CLP and ALI. The role of gut microbiota involved in the efficacy of JHD was evaluated by 16S rRNA sequencing and fecal microbiota transplantation (FMT). Translocation of intestinal *Escherichia coli* (*E. coli*) to lungs after CLP was verified by qPCR and in vivo-imaging. Intestinal permeability was analyzed by detecting FITC-dextran leakness. Junction proteins were evaluated by Western blotting and immunofluorescence.

**Results:** JHD treatment remarkably increased survival rate of septic mice and alleviated sepsis-associated lung inflammation and injury. FMT suggested that the protective role for JHD was mediated through the regulation of gut microbiota. We further revealed that JHD administration partially restored the diversity and configuration of microbiome that was distorted by CLP operation. Of interest, the intestinal bacteria, *E. coli* particularly, was found to translocate into the lungs upon CLP *via* disrupting the intestinal mucosal barrier, leading to the inflammatory response and tissue damage in lungs. JHD impeded the migration and hence lung accumulation of intestinal *E. coli*, and thereby prevented severe ALI associated with sepsis. This effect is causatively related with the ability of JHD to restore intestinal barrier by up-regulating tight junctions.

**Conclusion:** Our study unveils a mechanism whereby the migration of gut bacteria leads to sepsis-associated ALI, and we demonstrate the potential of JHD as an effective strategy to block this bacterial migration for treating sepsis and the associated immunopathology in the distal organs.

## Introduction

Sepsis is one of the leading causes of illness, disability and death in hospitalized patients with the mortality rate as high as 25%–30% (Fleischmann et al., 2016). It is estimated to affect at least 3 to 10 people per 1,000 per year in high-income countries, with most often between the ages of 18 and 35 (Perner et al., 2016). Sepsis can potentially cause the extensive inflammation and organ failure when the infection is unchecked and extend to the whole body. The lungs are the most vulnerable organs to sepsis-induced extensive inflammation, and over 50% of sepsis patients develop acute lung injury (ALI) or acute respiratory distress syndrome (ARDS) (Lelubre and Vincent, 2018). Currently, the treatment of sepsis mainly includes broad-spectrum antibiotic therapy, immune modulation and inflammation control, etc (Rhodes et al., 2017; van der Poll et al., 2017; Prescott and Angus, 2018), but the efficacy of these treatments is limited and the adverse effects are generally inevitable. Thus, it is urgently required to develop specific and efficient therapeutics with less or negligible side-effects to tackle sepsis and the accompanied complications.

Traditional Chinese medicine (TCM) has been recognized for its remarkable therapeutic efficacy and limited side effects. By targeting the major events during the onset of sepsis, TCM exhibits a network of regulatory function, ranging from inhibiting inflammatory responses, improving microcirculation, relieving gastrointestinal function to improving immune homeostasis (Fan et al., 2020). Among a few treatments for sepsis, Jinhong decoction (JHD), a TCM prescription, is distinguished by its potential effect to reduce the incidence, severity and complications of sepsis. JHD is made up of *Rheum palmatum L. (*Polygonaceae*; Rhei Radix et Rhizoma)*, *Sargentodoxa cuneata (Oliv.) Rehder and E.H.Wilson (*Lardizabalaceae*; Sargentodoxae Caulis)* and *Taraxacum mongolicum Hand.-Mazz. (*Asteraceae*; Taraxaci Herba)*, most of which have great anti-inflammatory, anti-infectious, anti-thrombotic and immunoregulatory roles. Longitudinal clinical and basic studies demonstrate that JHD might exert the sepsis-protecting functions by reducing endotoxin content, adjusting immune function, improving coagulation function and protecting organ function, but the exact mechanism is still unclear.

Previous studies on the pathogenesis of sepsis are mainly focused on hyperactivated inflammatory response, compromised immune regulatory function, increased cytokines production and unsuccessful clearance or inactivation of endotoxin (van der Poll et al., 2017). Nevertheless, increasingly emerging evidences have revealed the close relationship between gut microbiome dysbiosis and increased susceptibility to sepsis. Onset of sepsis not only alters the diversity and configuration of gut microbiome, but also affects the intestinal bioecosystem. Intestinal microenvironment is composed of biological barrier, namely gut microbiome, mucosal mechanical barrier and immune barrier. Perturbation of intestinal environment leads to gastrointestinal diseases and also systemic diseases likely due to the leakage of pathogenic agents through disrupted mucosal barrier (Muza-Moons et al., 2004). Maintaining the integrity of the intestinal epithelium is critical for the symbiosis in the gut, preventing the migration of intestinal bacteria to the distal organs such as lungs (Madara, 1990). Tight junction (TJ) is the most important components of the intestinal mechanical barrier (Grosheva et al., 2020). Destroy of TJ proteins, such as intact membrane proteins occludin, zonula occludens (ZO-1) and claudins would lead to the disruption of intestinal barrier and hence bacteria spreading and systemic infection and inflammation, the hallmarks of sepsis (Gu et al., 2011). Thus, retaining the function of intestinal barriers including mucosal mechanical barrier and biological barrier holds the key for healthy gut microecology.

Studies have shown that stress response after severe injury can cause intestinal mucosal barrier destruction, gut microbiome ecological imbalance and immune function decline, which in turn cause migration of intestinal bacteria or endotoxin release, triggering excessive inflammatory response and organ failure (Fay et al., 2017). These studies suggest that translocation of intestinal microbes into distal organs such as lungs might underlie severe compilations of sepsis. ALI is a most common cases induced by sepsis. The typical manifestation of ALI involves extensive pulmonary inflammation and injury, characterized by ample infiltration of proinflammatory cells including neutrophils, macrophages, and other immune cells like γδT cells, a subset recently proved to have a key role in mucosal immunity and respiratory diseases (Abraham, 2003). Of note, recent data indicate that intestinal factors might egress from the gut across disrupted intestinal barriers and enter the lungs *via* circulation, resulting in abnormal induction of inflammatory response and destroy of tissue homeostasis. In addition, bacteria and pro-inflammatory components such as LPS, bacterial DNA, and other products can spread into the circulation through the portal vein and liver, which subsequently reach the pulmonary through mesenteric lymph nodes. Thus, maintaining the so-called lung-gut balance is pivotal for the body, and gut dysbiosis as well as mucosal barrier collapse would predispose the individuals to a variety of airway diseases such as asthma, chronic obstructive pulmonary disease (COPD) and respiratory infections, etc (Budden et al., 2017). In healthy individuals, there is significant difference in microbiome composition between lung and intestine. By contrast, critically ill patients with severe sepsis or ARDS display the lung enrichment of gut-related microorganisms, corrugating with hyperinflammation and tissue damage in the respiratory system. Understanding how dysbiosis and abnormal microbial migration occur during sepsis onset is therefore essential for understanding the pathogenesis of the related diseases and developing the specific treatments.

By exploiting murine model of sepsis by CLP, we herein demonstrated that JHD remarkably alleviated the symptoms of sepsis and lung injury, which was related with its modulation of gut microbiome in sepsis mice. More importantly, our data show that sepsis-induced intestinal barrier disintergrity and the subsequent microbial translocation preceded the development of lung inflammation, which however was blocked by JHD *via* restoring intestinal barrier function. The findings thus provide a novel insight into the pathogenesis of sepsis-associated ALI and suggest a promising therapeutic for the related disorders.

## Materials and methods

### Reagents and antibodies

ZO-1 (Proteintech, 21773-1-AP); Occludin (Santa Cruz Biotechnology, sc-133256); Muc-1 (Abcam, ab15481); *β*-actin (Cell Signaling Technology, 3,700).

### Preparation and HPLC fingerprint of JHD

Clinically, Jinhong Decoction (JHD)is composed of *Rheum palmatum L. (*Polygonaceae*; Rhei Radix et Rhizoma)*, *Sargentodoxa cuneata (Oliv.) Rehder and E.H.Wilson (*Lardizabalaceae*; Sargentodoxae Caulis)* and *Taraxacum mongolicum Hand.-Mazz. (*Asteraceae*; Taraxaci Herba)*, and prepared by Shanghai University of Traditional Chinese Medicine. Briefly, 9 g *Rheum palmatum L.*, 30 g *Sargentodoxa cuneata (Oliv.) Rehder and E.H.Wilson* and 30 g *Taraxacum mongolicum Hand.-Mazz* were extracted twice with ethanol: water (95:5 V/V) and evaporated by a vacuum concentrator to 1.5 g crude drug/g extracts. Furthermore, the HPLC fingerprint of JHD extracts was established (Supplementary Figure S1 and Supplementary Table S1). A total of 11 well-separated peaks were identified in the fingerprint of JHD extracts at 254 nm, and the dominating metabolites were chlorogenic acid, caffeic acid, rhein and emodin, et al. Besides, the extracts of JHD were then prepared in phosphate buffer saline (PBS) for further study. *In vivo*, 0.1 ml/10 g body weight of JHD at 333.3 mg extracts/mL, which equals to 5 g crude drug/kg (converted from clinical dosage based on body surface area normalization), was administered intragastrically.

### Animal studies and cecal ligation-puncture (CLP) induced sepsis mouse model

Animal studies were performed according to the NIH Guide for the Care and Use of Laboratory Animals, and were approved by Animal Care and Use Committee of Nanjing University of Chinese medicine. C57BL/6 mice of 6–8 weeks were housed under pathogen-free conditions on a 12/12 h light/dark cycle with free access to food and water. CLP surgery is conducted as described previously (Rittirsch et al., 2009). Briefly, the mice were weighed and subjected to isoflurane gas anesthesia. A 2-cm longitudinal incision was made with a scalpel on the left side of the midline of the abdominal skin of mice, and the peritoneal cavity was further opened with small scissors. The cecum was ligated, punctured and then the peritoneum, fascia, and abdominal muscles were closed with sutures. Mice in the Sham group were treated with cecal manipulations without ligation and puncture. After surgery, 37°C prewarmed PBS (5 ml/100 g mouse body weight) were subcutaneously injected for postoperative resuscitation, followed by buprenorphine (0.05 mg/kg body weight) injection for postoperative analgesia.

### Fecal microbiota transplantation (FMT)

FMT was carried out according to the established protocol (Kim et al., 2020). Briefly, 6–8 weeks male C57BL/6 mice were divided into the donator and receiver goups. The donators were fed with JHD or the vehicle (PBS). After CLP surgery, intestinal contents were collected under sterile conditions 3 days after feeding. The collected intestinal contents of donator in each group were suspended 100 mg in 1 ml sterile PBS, which were then fully stirred for 10s, centrifuged at 800 g for 3 min to collect supernatant as graft material. Fresh graft material was prepared within 10 min before intragastric administration on the day of transplantation to prevent changes in bacterial composition. Thirty minutes after CLP operation in receiver, fresh graft material (100 μL/mouse/day) was intragastrically administrated. Two days later, the mice were euthanized and samples were collected for further experiments.

### Intestinal permeability analysis

Intestinal permeability was assessed using an FITC- dextran tracer (40 kDa, 20 ml/kg body weight; Chondrex). Specifically, mice were intragastrically administrated with 0.4 ml of FITC-Dextran before euthanizing. Blood samples were collected and allowed to clot for 30 min. Samples were then centrifuged for 90 s at 6,000 g. FITC-dextran concentration was determined using fluorophotometer at an excitation wave length of 488 nm and emission wave length of 520 nm (Vijay-Kumar et al., 2007)。

### Quantitative real-time PCR

Total RNA was extracted from mouse colon tissue, lung tissue using or intestinal content using RNAprep Pure Tissue Kit (TIANGEN) and Trizol reagent (Invitrogen) respectively. cDNA was generated using an HiScript II One Step RT-PCR Kit (Vazyme, P612-01). SYBR Green PCR Master Mix (TOYOBO) was used to quantify mRNA levels. The reactions were performed in triplicate, and relative mRNA expression was calculated using the 2^−△△Ct^ method. For cytokines or *E. coli*, relative levels of mRNAs were normalized with *Actb* or *universal bacteria* expression. Primers applied are listed in Supplementary Table S2.

### Flow cytometry analysis of cells in bronchoalveolar lavage fluid (BALF)

BALF was collected from mice at 3,000 rpm and centrifuged for 4 min. The supernatant was discarded and cell pellet was resuspended. BALF cells were stained with fluorescent-conjugated primary antibodies at 4°C for 30 min. Antibodies used were PE-cyanine 7-CD45 (Invitrogen), PE-TCRγδ (BioLegend) and FITC-CD3ε (Multi Science) for γδT cells; FITC-Ly6G (Invitrogen) and PE-CD11b (Invitrogen) for neutrophils. After being washed for three times, cells were suspended in 500 μl cell staining buffer for FACS analysis. γδT and neutrophils were defined as CD45^+^CD3^+^TCRγδ^+^ and Ly6G^+^CD11b^+^ lymphocytes, respectively. To detect γδT17 cell subset, cells were cultured with PMA (5 ng/ml) and BFA (1 ng/ml) at 37°C with 5% CO_2_ for 6 h. After washing, the cells were stained with FITC-CD3ε and PE-TCRγδ. The cells were infiltrated with Cytofix and stained with APC-IL-17A (Cat#17–7,177–81, Invitrogen). Flow cytometry was performed on Gallios (Beckman Coulter, United States of America) and FlowJo 10.4 (TreeStar, Ashland, OR) was used for analysis.

### Histology and immunohistochemistry studies

Tissue samples were fixed in 4% (w/v) formalin, dehydrated, embedded in paraffin, and then stained with hematoxylin and eosin (H&E staining) or the desired antibody (Muc-1, ZO-1). The specimens were observed under a Leica inverted microscope DMi8 and photographed. Five fields were observed for each section. Images were processed using NIH ImageJ software.

### 16S rRNA sequencing

16S ribosomal RNA (rRNA) sequencing was performed as previously described (Darnaud et al., 2018). Microbial DNA was extracted from fecal samples using the E. Z.N.A.^®^ Soil DNA Kit (Omega Bio-tek, Norcross, GA, U.S.) according to manufacturer’s protocols. The V4-V5 region of the bacteria 16S ribosomal RNA gene were amplified by PCR (95°C for 2 min, followed by 25 cycles at 95°C for 30 s, 55°C for 30 s, and 72°C for 30 s and a final extension at 72°C for 5 min) using primers 515F 5′-barcode- GTGCCAGCMGCCGCGG)-3′ and 907R 5′-CCGTCAATTCMTTTRAGTTT-3′, where barcode is an eight-base sequence unique to each sample. PCR reactions were performed in triplicate 20 μl mixture containing 4 μl of 5 × FastPfu Buffer, 2 μl of 2.5 mM dNTPs, 0.8 μl of each primer (5 μM), 0.4 μl of FastPfu Polymerase, and 10 ng of template DNA. Amplicons were extracted from 2% agarose gels and purified using the AxyPrep DNA Gel Extraction Kit (Axygen Biosciences, Union City, CA, U.S.) according to the manufacturer’s instructions. Purified PCR products were quantified by Qubit^®^3.0 (Life Invitrogen) and every 24 amplicons whose barcodes were different were mixed equally. The pooled DNA product was used to construct Illumina Pair-End library following Illumina’s genomic DNA library preparation procedure. Then the amplicon library was paired-end sequenced (2 × 250) on an Illumina MiSeq platform (Shanghai BIOZERON Co., Ltd.) according to the standard protocols. The raw reads were deposited into the GenBank database (Accession Number: SUB12419492).

### Bioinformatic analysis

Raw fastq files were first demultiplexed using in-house perl scripts according to the barcode sequences information for each sample with the following criteria: i) The 250bp reads were truncated at any site receiving an average quality score <20 over a 10 bp sliding window, discarding the truncated reads that were shorter than 50bp. ii) Exact barcode matching, two nucleotide mismatch in primer matching, reads containing ambiguous characters were removed. iii) only sequences that overlap longer than 10 bp were assembled according to their overlap sequence. Reads which could not be assembled were discarded. Passed sequences were dereplicated and subjected to the DADA2 algorithm (QIIME two recommended) to identify indel-mutations and substitutions. The trimming and filtering were performed on paired reads with a maximum of two expected errors per read (maxEE = 2). After merging paired reads and chimera filtering, the phylogenetic affiliation of each 16S rRNA gene sequence (herein called RSVs) was analyzed by uclust algorithm (http://www.drive5.com/usearch/manual/uclust_algo.html) against the silva (SSU138.1)16S rRNA database using confidence threshold of 80%. Sequences were clustered into operational taxonomic units (OTUs) at 100% similarity (identical) using the Deblur denoising algorithm, which removes noise due to sequencing error. The phylogenetic affiliation of each 16S rRNA gene sequence was analyzed by uclust algorithm (http://www.drive5.com/usearch/manual/uclust_algo.html) against the silva (SSU138.1)16S rRNA database using confidence threshold of 80%.

Beta diversity analysis was performed using UniFrac to compare the results of the principal component analysis (PCA) using the community ecology package, R-forge (Vegan 2.0 package was used to generate a PCA figure). By using iterative algorithm in QIIME1.8 software, PCoA analysis was carried out based on the beta-diversity index matrix obtained by the abovementioned calculation, and the main coordinate with the largest contribution rate was selected to draw the coordinate diagram. For identification of biomarkers for highly dimensional colonic bacteria, LEfSe (linear discriminant analysis effect size) analysis was done. Kruskal–Wallis sum-rank test was performed to examine the changes and dissimilarities among classes.

### Bacterial load assay

As previously described (Santos et al., 2020), blood and lung tissues were collected at a specified time point after CLP surgery. The homogenate of blood or lung tissue is diluted by multiplication. Subsequently, 10 μL of diluent was added to nutrient agar plate to be cultured for 18 h, and CFU counting was performed.

### *E. coli* migration experiment

*E. coli* (DH5α) were incubated with 1,1′-Dioctadecyl-3,3,3′,3′-Tetramethylindocarbocyanine Perchlorate (DiL) fluorescent dye (Invitrogen) for 30 min. The labeled *E. coli* (5 × 10^5^ CFU/mice) were then given rectally 2 h after CLP.

### Pathological analysis

Pathological analysis was conducted according to a standard histological lung injury scoring system (Supplementary Table S3), which includes the assessments from both laboratory researchers and pathologists. The scores were then calculated following the formula: total score = [(20 × A) + (14 × B) + (7 × C) + (7 × D) + (2 × E)]/(number of fields × 100) to make the final grades. For colon tissue samples: according to the five-grade scoring principle (Supplementary Table S4).

### Statistical analysis

Data were presented as the mean ± standard deviation (SD) of results in at least three independent experiments unless otherwise stated. Statistical significance of differences was assessed with Student’s t-test for two groups or one-way ANOVA for multiple groups. Differences were considered significant when *p* < 0.05. Data were calculated using GraphPad Prism 8.3.1.

## Results

### JHD confers the protection of mice against CLP-induced sepsis

**FIGURE 1:**
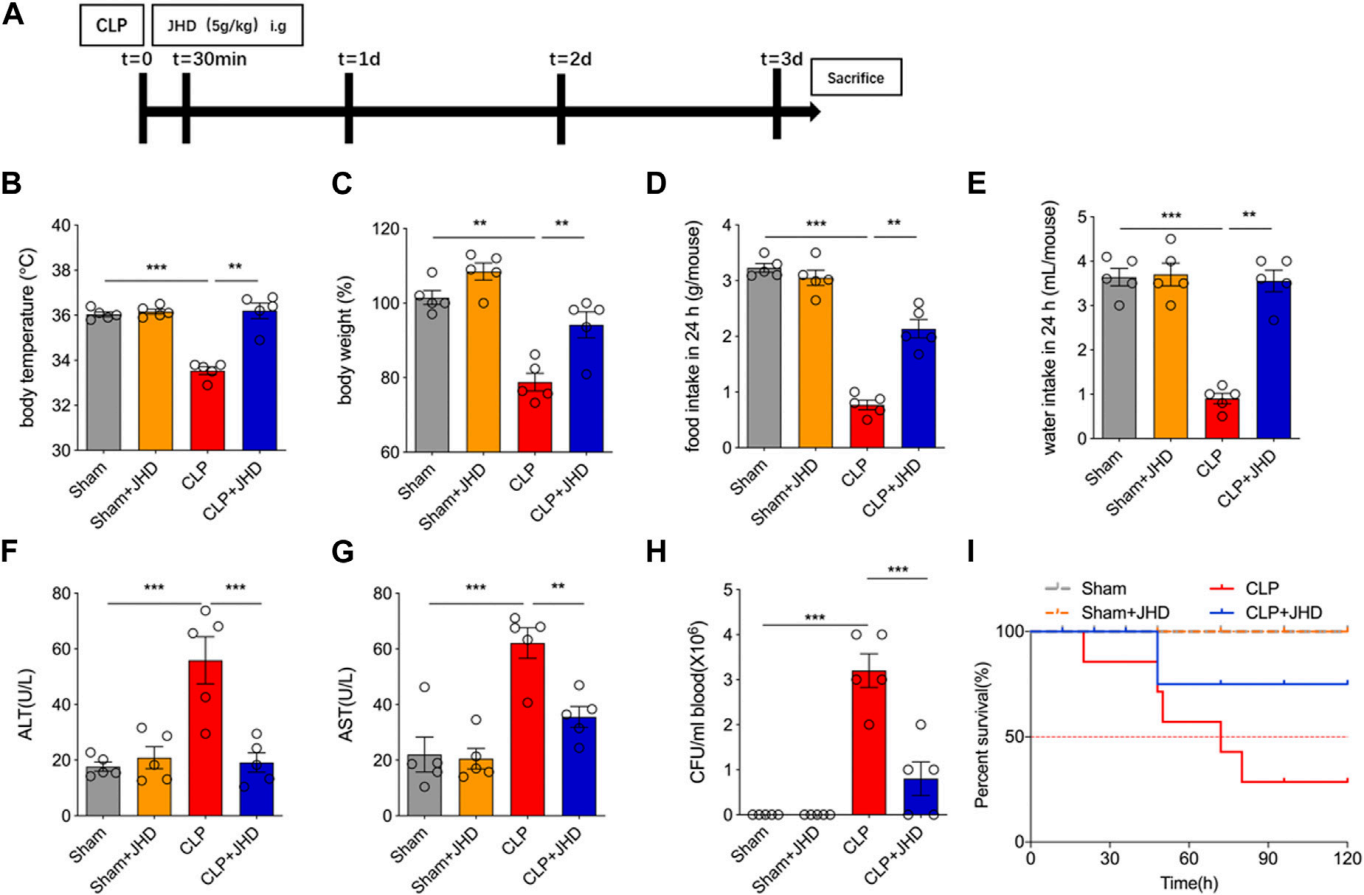
JHD attenuated symptoms of CLP-induced sepsis in mice. **(A)** Flow chart of model establishment and JHD administration; Sepsis-characterized symptoms including **(B)** body temperature, **(C)** body weight, **(D)** food intake, and **(E)** water intake; Serum level of **(F)** ALT and **(G)** AST; **(H)** Bacterial load in circulation; **(I)** Survival rates. (mean ± SD, *n* = 5; ^*^ *p* < 0.05, ^**^ *p* < 0.01, ^***^ *p* < 0.001).

In order to study the potential therapeutic efficacy of JHD in sepsis, we applied CLP to induce sepsis in mice, and JHD was administered 30 min post surgery (Figures 1A). The results showed that compared with the Sham group, body weight, body temperature, diet and water intake of mice in the CLP group were significantly reduced, and JHD could reverse these changes (Figures 1B–E). Furthermore, JHD markedly reduced the serum levels of alanine aminotransferase (ALT) and aspartate aminotransferase (AST) in sepsis mouse model, the indicators of liver damage (Figures 1F,G). Enumeration of bacterial loads by the dilution plate assay indicated that the number of bacteria in blood of sepsis mice was markedly increased, which however was significantly reduced by JHD administration (Figure 1H). Besides, JHD markedly increased the survival rate of sepsis mice (Figure 1I). Collectively, JHD showed potent efficacy in alleviating CLP-induced experimental sepsis, analogous to its clinical therapeutic effects.

### JHD reduces lung inflammation and injury associated with sepsis

**FIGURE 2:**
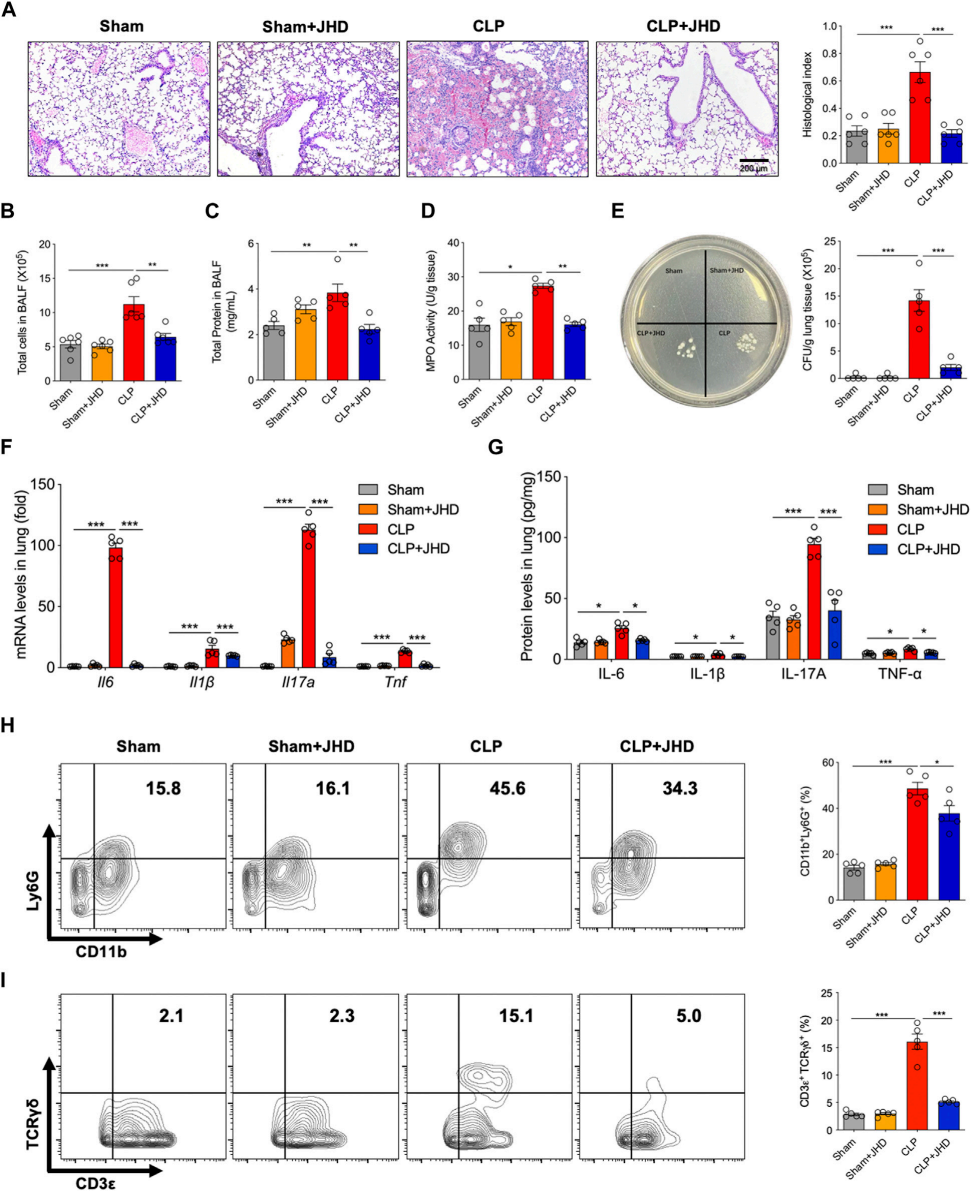
JHD alleviates sepsis-induced lung injury. **(A)** H&E staining of lung tissues and the correlated pathological scores (magnification: ×400, scale bar = 200 μm); **(B)** Total cells infiltrated and **(C)** total protein leaked in BALF; **(D)** MPO activity in lung tissue; **(E)** Bacterial loads in lung tissues; **(F)** qPCR assay of *Il6*, *Il1β*, *Il17a* and *Tnf* levels in lung tissues; **(G)** ELISA assay of IL-6, IL-1β, IL-17A and TNF-α levels in lung homogenate; Flow cytometry analysis of **(H)** CD11b^+^Ly6G^+^ neutrophils and **(I)** CD3ε^+^TCRγδ^+^ γδ T cells in BALF (%). (mean ± SD, *n* = 5–6;^*^ *p* < 0.05, ^**^ *p* < 0.01, ^***^ *p* < 0.001).

We next evaluated the protective efficacy of JHD on sepsis-induced ALI. As shown in Figure 2A, H&E staining of lung sections showed that JHD alleviated the infiltration of inflammatory cells into alveolar wall and decreased transparent film generation and fibrin bundles production in sepsis mice. It also reduced total cell number, total protein content in BALF, and MPO activity of lung tissues, indicative of lung inflammation and injury (Figures 2B–D). In addition, JHD treatment significantly reduced bacterial burdens in lung homogenate of sepsis mice (Figure 2E). The levels of proinflammatory cytokines including IL-6, IL-1β, Il-17A and TNF-α, as detected by quantitative PCR or enzyme-linked immunosorbent assay (ELISA), were decreased in lung tissue of sepsis mice upon treatment of JHD, as compared with their counterpart with no treatment (Figures 2F,G). In line with the remarkable inhibition of IL-17A production by JHD, we showed that the cytokine-generating immune cells such as neutrophils (CD11b^+^Ly6G^+^) and γδT (TCRγδ^+^CD3ε^+^) cells were up-regulated in the model group and profoundly suppressed by JHD treatment (Figures 2H, I).

### JHD regulates intestinal microbiome composition in sepsis mouse models

**FIGURE 3:**
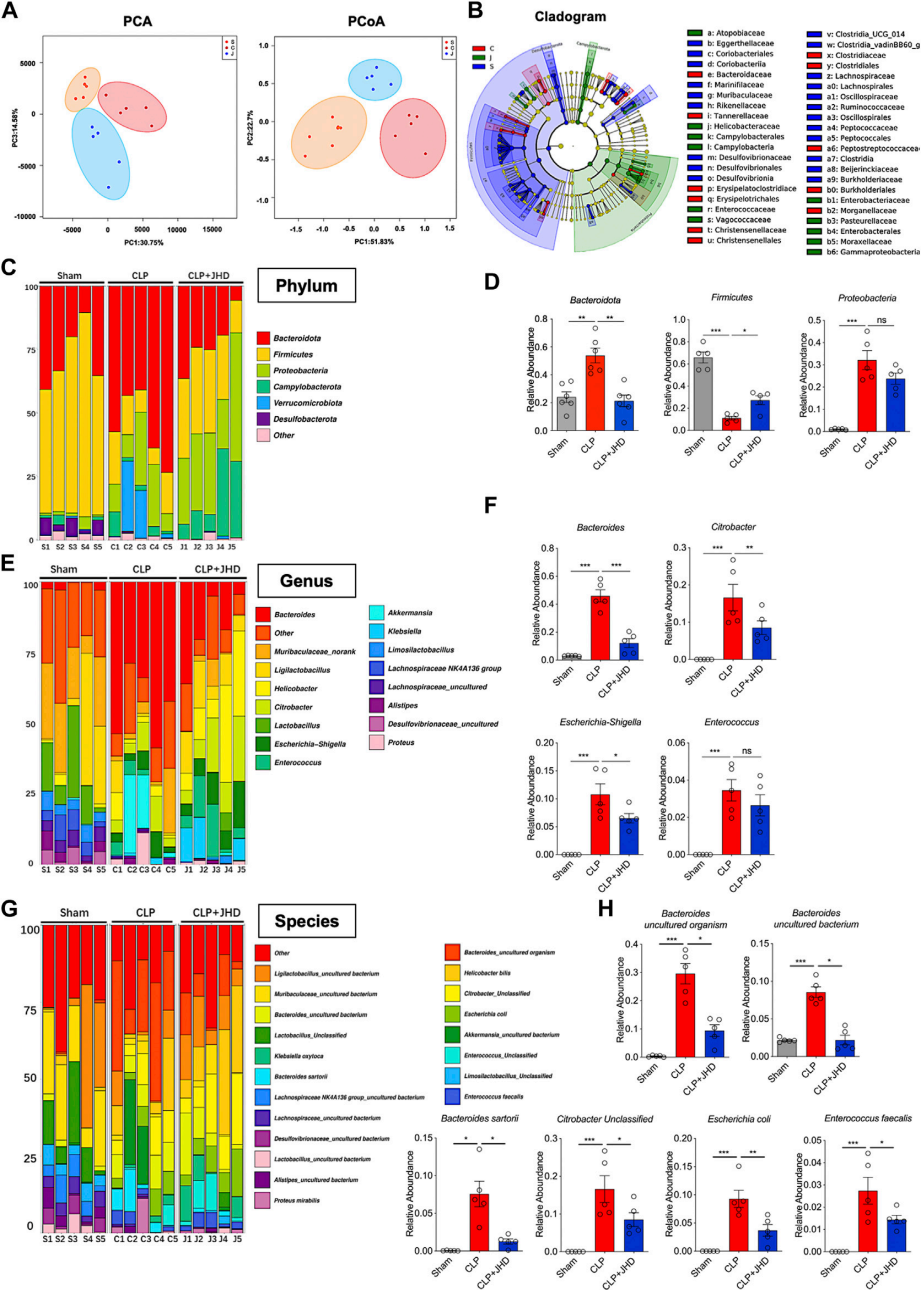
JHD modulates the composition of intestinal microbiome in sepsis mice. **(A)** Principal Component Analysis (PCA) and Principal Co-ordinates Analysis (PCoA) of gut microbiome. Orange, red and blue represents the sham (S), CLP (C) and CLP + JHD (J) groups, respectively; **(B)** Linear discriminant analysis Effect Size (LefSE) analysis. Deep blue, red and green represents the sham (S), CLP (C) and CLP + JHD (J) groups, respectively; **(C)** The composition of gut microbiome at phylum level, and **(D)** the representative bacteria with significant changes between the indicated groups (*Bacteroidota*, *Firmicutes* and *Proteobacteria*). **(E)** The composition of gut microbiome at genus level, and **(F)** the representative bacteria with marked changes (*Bacteroides*, *Citrobacter*, *Escherichia-Shigella, Enterococcus*); **(G)** The composition of gut microbiome at species level, and **(H)** the representative bacteria with significant change (*Bacteroides*, *Citrobacter*, *Escherichia coli* and *Enterococcus faecalis*). (mean ± SD, *n* = 5; ^*^ *p* < 0.05, ^**^ *p* < 0.01, ^***^ *p* < 0.001).

Increasingly emerging evidences have demonstrated a close link between sepsis and gut microbiome. In order to prove that JHD may have a potential role in regulating the dysbiosis associated with sepsis, we exploited 16S rRNA sequencing for analyzing the diversity and structure of gut microbiome. The results showed that *β* diversity, representing the heterogeneity of species composition among different communities, was greatly different between Sham, CLP and CLP + JHD groups (Figure 3A), as reflected by PCA and PCoA. LefSE analysis showed that the intestinal microbiome after CLP surgery was dominated by *Clostridiacease*, Christensenellaceae, *Desulfobacterota* and other genera bacteria, and JHD treatment reduced this alteration (Figure 3B). Furthermore, the barplot species composition analysis was performed to study the differences in relative abundance of intestinal microbiome in each group of mice. It was revealed that the intestinal microbiome at phylum level was mainly composed of *Bacteroidetes*, *Firmicutes*, *Proteobacteria*, *Verrucomicrobia* and *Deferribacteres*. In the CLP-induced sepsis mice, the relative abundance of *Bacteroidetes* was increased while that of *Firmicutes* was decreased. Treatment of JHD profoundly reversed the trend by down-regulating the frequency of *Bacteroidetes* and elevating that of *Firmicutes* (Figures 3C,D). At the genus level, the relative abundance of *Bacteroidetes*, *Citrobacter*, *Enterococcus* and *Escherichia Shigella* was increased in sepsis mice, which was significantly reduced by JHD treatment (Figures 3E, F). Analysis on gut microbiome at species level further indicated that JHD down-regulated the relative abundance of *Escherichia coli*, *Enterococcus faecalis* and *Citrobacter* (Figures 3G,H). Thus, these data demonstrated that JHD significantly changed the gut microbiota composition in sepsis mice and improved the dysbiosis linked with sepsis.

### The protective effect of JHD is mediated through gut microbiome

**FIGURE 4:**
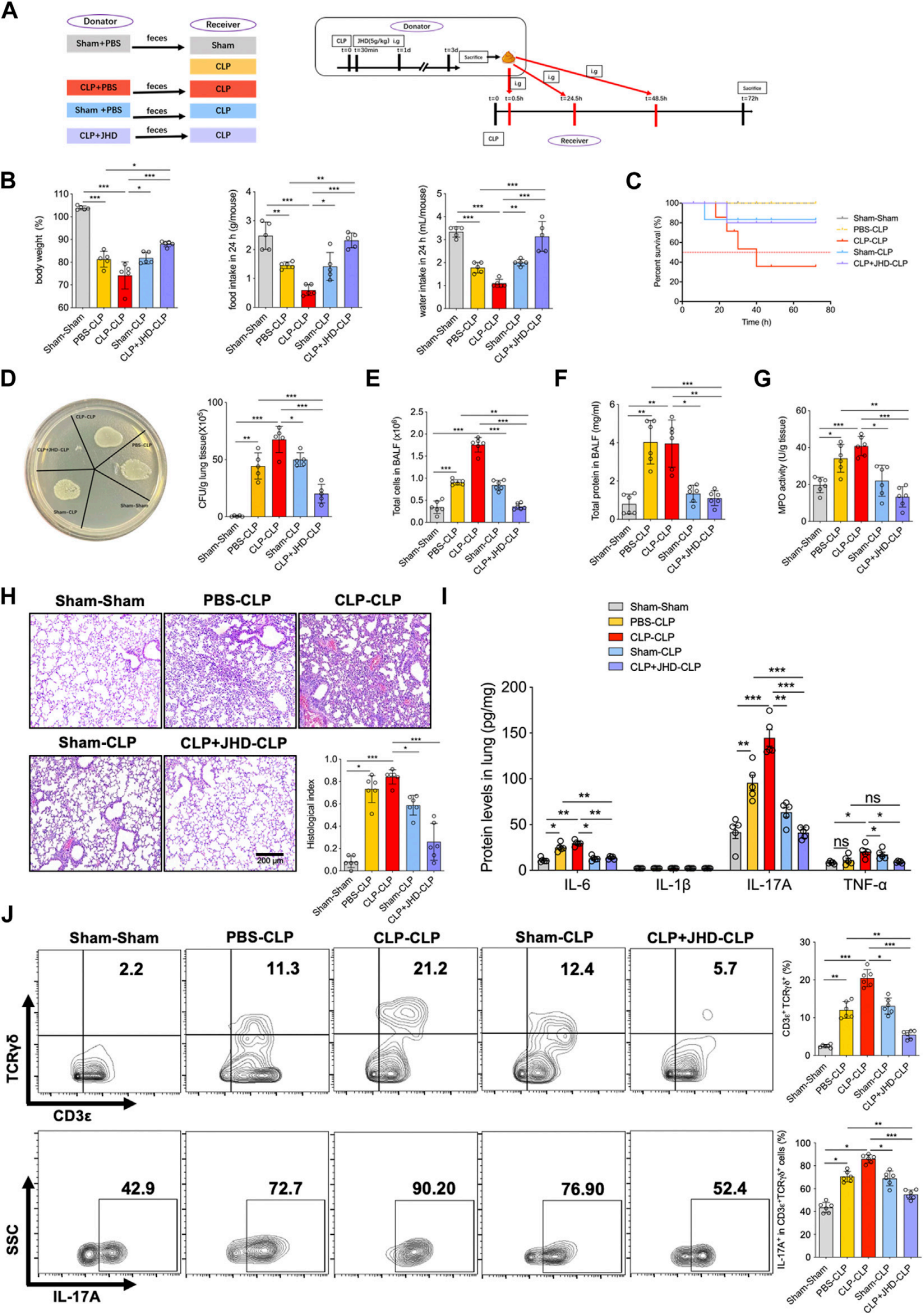
JHD confers the protective effect on sepsis-associated ALI *via* FMT. **(A)** Experimental flow chart; **(B)** Body weight, food consumption and water intake; and **(C)** Survival rates of mice with different FMT; **(D)** Bacterial load in lungs; **(E)** Total cell counts, and **(F)** protein leakage in BALF; **(G)** MPO activity in lungs. **(H)** H&E staining of lung sections and correlated pathological scores (magnification: ×400, scale bar = 200 μm); **(I)** ELISA assays of the levels of inflammatory cytokines in lung homogenates; **(J)** CD3ε^+^TCRγδ^+^ γδT (%) and IL-17A^+^ γδT (%) in BALF were analyzed by flow cytometry. (mean ± SD, n = 6, ^*^ *p* < 0.05, ^**^ *p* < 0.01, ^***^ *p* < 0.001).

Given the above finding about the profound function of JHD in regulating gut microbiome, we then explored whether JHD conferred the protective effect through gut microbiome. To this end, FMT experiment were carried out and experimental design was depicted in Figure 4A. Firstly, we observed that compared with the model group (PBS-CLP) and the model groups with Sham FMT (Sham-CLP), the sepsis mice that received the gut microbiome from CLP mice (CLP-CLP), demonstrated more severe symptoms, as demonstrated by body weight, diet and water intake (Figure 4B). Strikingly, the sepsis mice receiving the microbiome from JHD-treated CLP mice (CLP + JHD-CLP) showed a significant improvement in these disease manifestations (Figure 4B). More critically, the survival rate of CLP + JHD-CLP was remarkably heightened compared with those receiving fecal microbes of non-treated sepsis mice (CLP-CLP) (Figure 4C), substantiating the gut microbiome-dependency of JHD effect.

Since JHD treatment significantly improved lung pathology and impeded the gut-lung bacterial translocation upon CLP operation, we then sought to address whether the pulmonary protective effect of JHD would be transferred by FMT. Indeed, our data showed that bacterial loads in lung tissue of CLP + JHD-CLP group was significantly reduced comparing with that in CLP-CLP group (Figure 4D). Associated with this, total cells infiltration and protein leaked in BALF, as well as lung MPO activity were significantly reduced in CLP + JHD-CLP relative to CLP-CLP groups of mice (Figures 4E–G). Pathological changes of lungs were consistently attenuated in sepsis mice taking up the gut microbes of JHD-conditioned mice (Figure 4H). Moreover, the transfer of fecal bacteria from JHD-CLP mice significantly reduced the levels of proinflammatory cytokines including IL-17A (Figure 4I), and the percentages of γδT and γδT-17 cells were in parallel (Figure 4J). Together, these results indicated that JHD was able to restore gut microbiota in sepsis mice, and prevent sepsis and associated ALI through FMT.

### The gut bacteria in sepsis mice migrate to the lungs through damaged intestinal barrier

**FIGURE 5:**
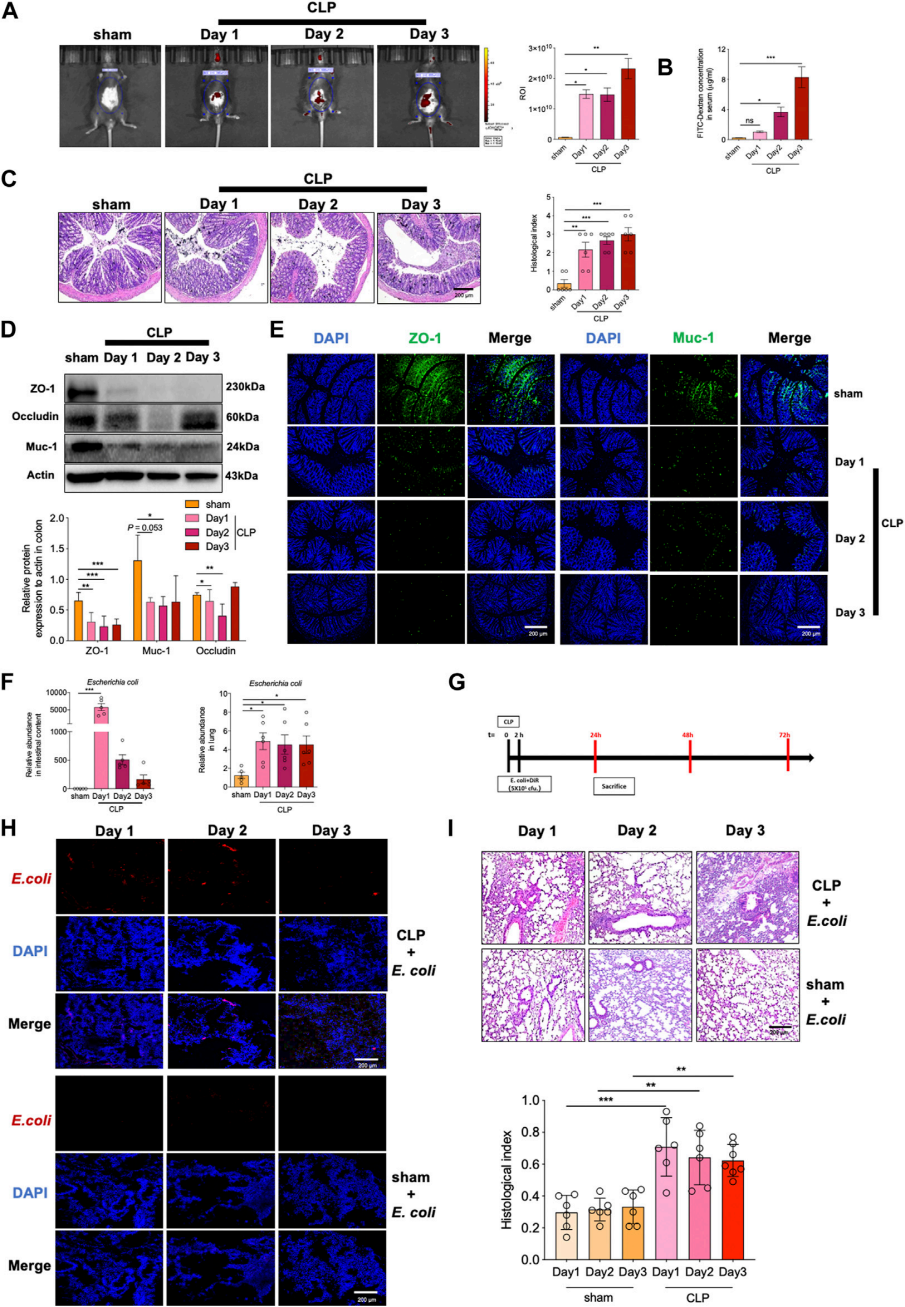
Sepsis causes impaired intestinal barrier and gut-lung translocation of *E. coli* to induce lung pathology. Intestinal permeability was determined by measuring **(A)** fluorescence intensity of FITC-dextran and **(B)** serum FITC-dextran concentration. **(C)** H&E staining of colon tissues and correlated pathological scores (magnification: ×400, scale bar = 200 μm); **(D)** Immunoblotting of ZO-1, Occludin and Muc-1; **(E)** immunofluorescent staining of ZO-1 and Muc-1; **(F)** Relative abundance of **(E)** *coli* in intestinal contents and lung tissues; **(G)** DiL-labeled *E. coli* was given rectally to mice (10^6^ CFU per mouse), and 2 h later, the mice were subjected to CLP; **(H)** Frozen section of lung tissues was detected for DiL-labeled *E. coli*; **(I)** H&E staining of lungs and correlated pathological scores (magnification: ×400, scale bar = 200 μm) (mean ± SD, *n* = 5 or 6; ^*^ *p* < 0.05, ^**^ *p* < 0.01, ^***^ *p* < 0.001).

Disruption of intestinal barrier integrity may lead to the translocation of intestinal bacteria that are normally not pathogenic and cause the diseases in distal organs such as lungs (Chopyk and Grakoui, 2020). To investigate the intestinal barrier function during sepsis, we then instilled FITC-labeled dextran intragastrically to detect intestinal permeability. *In vivo* imaging showed that CLP surgery substantially increased intestinal permeability with the most effect at the third day (Figure 5A). Serum concentration of FITC-dextran was consistently up-regulated after CLP surgery (Figure 5B). More severe pathology, as demonstrated by extensive loss of cup-shaped fine wrapping and most lesions of the crypt, were observed in CLP mice compared with the Sham group (Figure 5C). In addition, the levels of ZO-1 and Occludin, the two major junction proteins, and that of the mucin protein Muc-1were significantly decreased in colon tissues of sepsis mice (Figure 5D). Lower level of Muc-1 and ZO-1 were further confirmed by immunofluorescence staining (Figure 5E). We thus proposed that the intestinal barrier was remarkably impaired in sepsis mice.

Since disruption of barrier integrity was frequently associated with abnormal bacterial translocation, we then assessed the enrichment of gut bacteria in lungs. In accordance with the above data by microbial sequencing, the relative abundance of *E. coli* was significantly up-regulated in sepsis mice (Figure 5F). *E. coli* is known as an opportunity pathogen that may induce lung pathology after migrating into the airway. To test this possibility, we then instilled DiL-labeled *E. coli* rectally and detected its accumulation in lung tissues of sepsis mice (Figure 5G). As a result, the enrichment of *E. coli* was evidently detected in lung tissues of CLP mice, whereas sham group of mice displayed negligible level of *E. coli* migrating into the lungs (Figure 5H). Further pathological analysis demonstrated that this bacterial translocation induced exacerbated lung inflammation and injury in CLP mice relative to Sham treated mice (Figure 5I). Together, the data indicated that the integrity and function of intestinal barriers were significantly disturbed upon sepsis, leading to boosted bacterial translocation from damaged gut to lungs and subsequently causing pulmonary inflammatory pathology.

### JHD prevents gut bacterial translocation through restoring intestinal barrier function

**FIGURE 6:**
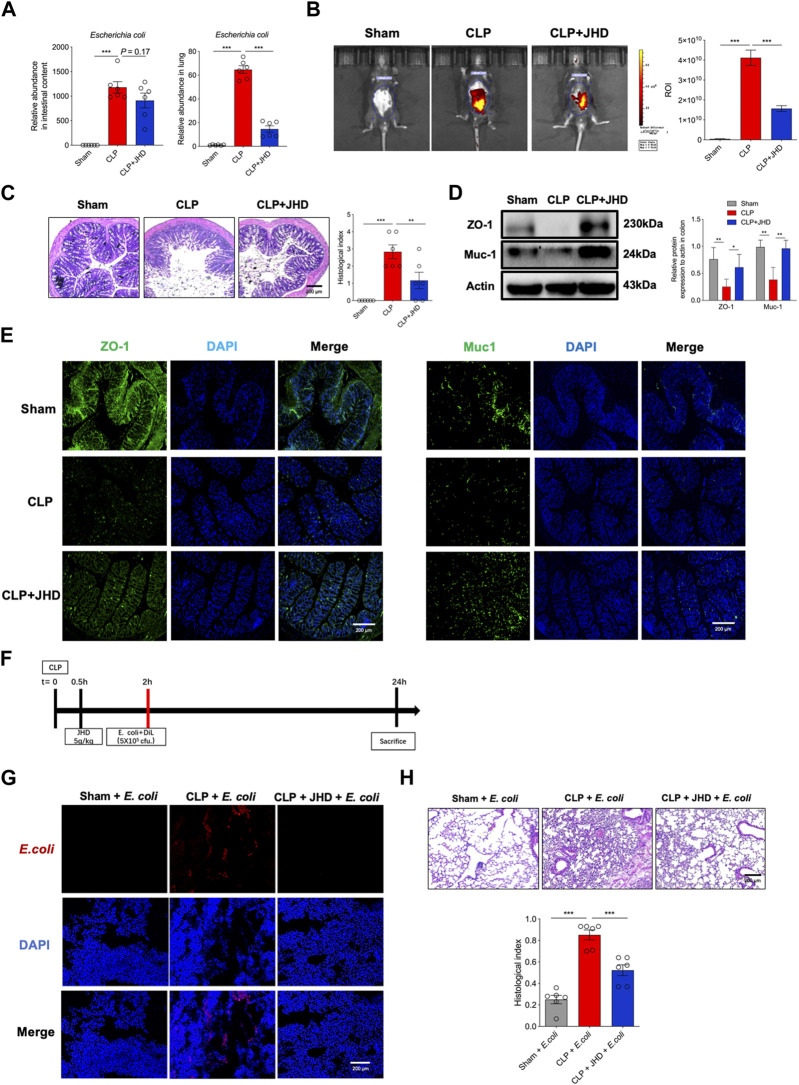
JHD improves intestinal mucosal barrier and prevents gut bacterial translocation for inducting lung inflammation. **(A)** Relative abundance of *E. coli* in intestinal contents and lung tissues; **(B)**Intestinal barrier permeability measured by *in vivo* imaging of FITC-dextran; **(C)** H&E staining of colon tissues and correlated histological analysis (magnification: ×400, scale bar = 200 μm); ZO-1 and Muc-1 in colon tissues were determined by **(D)** Western blotting and **(E)** immunofluorescence; **(F)** Experimental flow chart; **(G)** DiL-labeled *E. coli* in frozen lung sections; **(H)** H&E staining of lung and correlated pathological scores (magnification: ×400, scale bar = 200 μm) (mean ± SD, *n* = 6;^*^ *p* < 0.05, ^**^ *p* < 0.01, ^***^ *p* < 0.001).

As portrayed in Figure 6A, JHD treatment cause a profound reduction in *E. coli* enrichment in lungs of sepsis mice, whereas no significant change was observed in intestinal content. To address whether this effect was due to the blockade of bacterial migration *via* restoring intestinal barrier function, we then exploited FITC-dextran for *in vivo* imaging. The results showed that the fluorescent signals were markedly weakened in JHD-treated mice relative to their counterparts with no treatment, supporting the improved intestinal integrity by JHD treatment (Figure 6B). Pathological analysis also indicated that JHD administration prevented loss of goblet cells in the gut (Figure 6C). Western blotting and immunofluorescence revealed that JHD restored the expression of ZO-1 and Muc-1 in colon tissues in sepsis mice (Figures 6D,E). To further test whether JHD could prevent the migration of *E. coli* and induce the inflammatory pathology in lungs, DiL-labeled *E. coli* was given rectally in CLP mice (Figure 6F). Remarkably, abundant DiL-labeled *E. coli* was detected in the lungs of sepsis mice but not JHD-treated ones (Figure 6G). Associated with this, the symptoms of *E. coli*-induced lung injury were significantly attenuated in JHD-treated CLP mice (Figure 6H). Thus, the data indicated that JHD prevented intestinal bacteria translocation *via* improving gut barrier integrity and thereby alleviated lung inflammatory response and tissue damage.

## Discussion

Sepsis is a severe infectious and inflammatory disorder accompanied by acute organ injury, posing the great threat for human health worldwide. It is generally thought as the consequence of maladjustment of host’s response to infection and the ability to control inflammation. Due to high incidences and mortality, the discovery of effective and specific treatments is urgently needed to prevent or control sepsis and the associated symptoms such as ALI (Cecconi et al., 2018). JHD is a TCM prescription capable of reducing endotoxin, sterilization and regulating immune function. Its potential active ingredients include rhein, emodin, chlorogenic acid, caffeic acid, etc, which have been reported to have anti-inflammatory, immunoregulatory and tissue-protective function (Li et al., 2016; Yin et al., 2016; Wang et al., 2017; Wu et al., 2020). Currently JHD has been used in clinical practice to treat sepsis and associated organ injury, but the mechanism is yet to be explored. We firstly conducted preliminary experiments to explore the protective effect of JHD at 2.5, 5 and 10 g/kg. Results suggested that 5 g/kg, converted from the clinical dosage, was potent while 2.5 g/kg only showed mild efficacy (data not shown). Considering of the 4R rules (reduce, refine, replace and responsibility), we performed the exploratory experiments in this study using a single dose of JHD at 5 g/kg. We demonstrate that JHD conferred a profound protection against sepsis and associated lung injury in mice. The effect was causatively related with the potency of JHD to rectify the gut dysbiosis caused by CLP surgery. More importantly, JHD treatment impeded the translocation of intestinal bacteria, namely *E. coli*, to the respiratory system and prevented pulmonary inflammation and injury. The restoration of intestinal mucosal barrier function and integrity was proved to underline this effect. Collectively, our data confirm the therapeutic potential of JHD in sepsis and associated ALI, which is of great clinical relevance for these deadly disorders with no cures. We also uncover a gut-lung crosstalk that is specified by intestinal microbes and might be a potential target for treating the distal tissue pathology caused by sepsis.

Intestinal tract is an important organ affected by trauma, burn, shock, hemorrhage and infection. Disturbances in intestinal microbiome and deregulation in intestinal epithelial cells and immune system would lead to gut damage, and subsequently infections and multiple organ dysfunction (Shimizu et al., 2018). Evidences have shown that patients with low diversity of gut microbiota and high relative abundance of pathogenic Gram-negative bacteria and *Enterococcus* are at higher risk of sepsis. Reciprocally, sepsis promotes gut microbiota disorder and hence forms a vicious cycle. It appears that changes in intestinal microbiota by sepsis precedes the onset of metabolic endotoxemia, widespread tissue inflammation and related diseases. The mechanism for this dysbiosis is not completely understood, but the disruption of tight junction and increased intestinal permeability are critically involved (Cani et al., 2008). Our current study showed that in the murine sepsis model, the diversity and configuration of gut microbiome were substantially altered upon CLP surgery. In particular, the relative abundance of *E. coli*, *Citrobacter* and *Enterococcus* was significantly increased, which however was repressed by JHD treatment. As is known, *E. coli*, belonging to proteobacteria, is traditionally thought as a conditional pathogenic bacterium. It may also cause intra-abdominal infections, urinary tract infections, and even sepsis when enters the circulation (Speer et al., 2020; Keshari et al., 2021). Interestingly, our 16S rRNA sequencing data also revealed that the abundance of gut *Bacteroidetes* was up-regulated while *Firmicutes* was down-regulated in CLP-induced sepsis. This appears to contradict with the reported relevance of the *Firmicutes/Bacteroidetes* (F/B) ratio to the gut dysbiosis and inflammatory pathology. The plausible reason for that might be due to the complexity of experimental materials and methods utilized, such as the mice lineage and culture, the septic surgery, the facet collecting and delivery, as well as the bacteria sequencing and analysis. These factors may cause the inconsistency in the sepsis-related microbiome. As is known, the genus of *Firmicutes* and *Bacteroidetes* are proficient in healthy human beings, but depleted in the patients with sepsis, suggesting a correlation between alteration in gut microbiota composition and sepsis outcome (Bassetti et al., 2020). Of interest, the microbiota composition of septic patients demonstrated to shift towards normalized following fecal microbiota transplantation (FMT) with a profound expansion in Firmicutes, accompanied with improved clinic manifestation (Li et al., 2015; Wei et al., 2016). The findings indicate that defective *Firmicutes* genus is likely related with the sepsis pathogenesis and can be corrected by microbiota normalization. As an additional support, a recent study reported that the abundance of *Firmicutes* was negatively correlated with inflammatory markers, such as IL-6, IL-18, HMGB-1, or CRP, or Th two cell (Li et al., 2015). *Bacteroidetes* is another common bacteria with the ability to actively alter the gut environment to their benefit (Wexler and Goodman, 2017), and resiliently localize in the presence of acute inflammation (Cullen et al., 2015). In line with our observation, studies have shown the presence of *Bacteroidetes* in post-sepsis lungs in mice and humans (Dickson et al., 2016). The relative abundance of *Bacteroides* was proved to parallel with the serum TNF-α concentration, predictive of patient mortality (Osuchowski et al., 2006). Thus, further studies are needed to dissect the contribution and/or the clinic implication of these two major gut bacteria in sepsis and related diseases. By FMT approaches, we proved that JHD-conditioned gut microbiota from sepsis mice markedly alleviates the severity of sepsis and ALI, suggesting that the protective effect of JHD was largely dependent on its modulation of intestinal microbes of sepsis mice. In addition to the disturbed gut microbiome, sepsis also causes impaired tight junction in the intestine and increased al barrier permeability, thereby facilitating the translocation of microbes to distal organs like lungs (van der Poll et al., 2021). In this study, we exploited the bacteria-tracing strategies to demonstrate that sepsis enhanced migration of intestinal bacteria, particularly *E. coli*, and caused severe lung inflammation and injury. This may provide an explanation for the report that there is a certain similarity in the pulmonary and intestinal microflora of sepsis mice (Dickson et al., 2016). Of interest, JHD exhibits the unique capability to block the migration of *E. coli* to the lungs, correlating the alleviated pulmonary inflammation and injury. Supportively, JHD potentially improve intestinal mucosal barrier integrity by increasing the expression of barrier-related proteins ZO-1 and Muc-1. We thus uncover an unappreciated mechanism underpinning JHD-mediated tissue-protecting function beyond its canonical anti-inflammatory role. Future studies are merited to dissect the contributions of the key active ingredient of JHD in these processes.

To identify the key effector cells targeted by JHD, we analyzed the proportion of major immune cells during acute lung inflammatory responses, such as neutrophils and γδT cells in BALF of sepsis mice. Intriguingly, we found that the proportion of γδT and neutrophils was significantly increased in sepsis mice, which was suppressed by JHD treatment. γδT cells represent a major immune cell population in the barrier sites characterized by the secretion of cytokine IL-17A. The up-regulation of γδT cells has been evidenced in a range of lung diseases such as viral pneumonia, asthma and lung cancer (Jin et al., 2019). Recent studies showed that *E. coli* entering the liver would promote the proliferation of liver γδT-17 cells in a CD1d lipid-dependent manner (Li et al., 2017; Xue et al., 2017). Likewise, respiratory viral infection induced the generation of γδT-17 cells *via* CD1d-mediated antigen presentation (Jensen et al., 2008). Our results showed that *E. coli* could migrate into the lung and subsequently promote lung inflammation in the sepsis model. This process may be related to the lipid metabolites of *E. coli*, which further through CD1d-medited antigen presentation to induce γδT-17 cells and lung pathology. The hypothesis however needs to be experimentally tested and confirmed.

In summary, our present study demonstrates the therapeutic effect of JHD on sepsis and the associated lung inflammation and injury, which is causatively related with its ability to rectify the gut dysbiosis, improve intestinal barrier integrity and hence impede the translocation of microbiome bacteria. The findings extend our understanding about the pathogenesis of sepsis-related ALI and suggest a promising therapeutic approach for the lethal disorders.

## Data Availability

The datasets presented in this study can be found in online repositories. The names of the repository/repositories and accession number(s) can be found below: NCBI GenBank [https://www.ncbi.nlm.nih.gov/genbank/], OQ026974 - OQ028103.
