# Supplementary material for: Jinhong decoction protects sepsis-associated acute lung injury by reducing intestinal bacterial translocation and improving gut microbial homeostasis
Source: Front Pharmacol. 2023 Apr 4;14:1079482. doi: 10.3389/fphar.2023.1079482 (PMC10110981; doi:10.3389/fphar.2023.1079482)
Supplement: Supplementary file 1 [file DataSheet1.PDF]

Supplementary Material

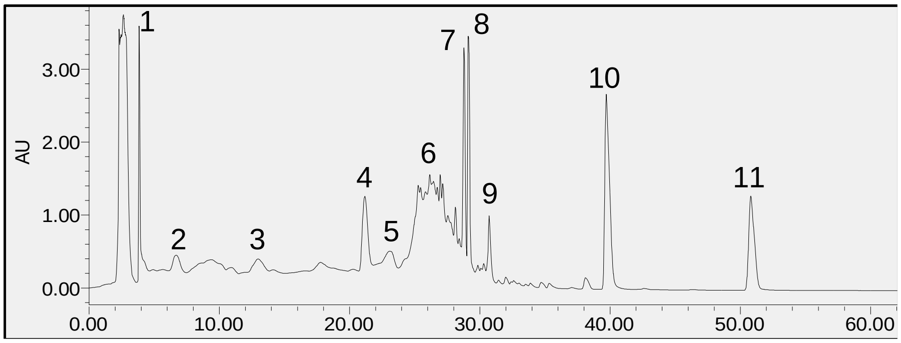

Supplementary Figure 1. 0.5 g JHD sample was precisely weighed and extracted with 10 mL methanol-water (70:30 V/V) under ultrasonication (100 kHz) for 20 min. Ten microliters of the prepared JHD sample or reference standard compounds including emodin, rhein, chlorogenic acid and caffeic acid were injected into HPLC system (Waters) for analysis. Chromatographic separations were performed on a C18 110A column (250 × 4.6 mm i.d., 5 μm, Waters) at 30℃. Samples were analyzed by a gradient method at a flow rate of 1mL/min and the mobile phase consisted of water containing 0.1% phosphoric acid (A, v/v) and acetonitrile (B) with the gradient of 10-20% B at 0-20 min, 20-40%

B at 20-25 min, 40-60% B at 25-55 min, 60-80% B at 55-65 min. A total of 11 well-separated peaks were identified in the fingerprint of JHD extracts at 254 nm (see **Supplementary Table 1**).

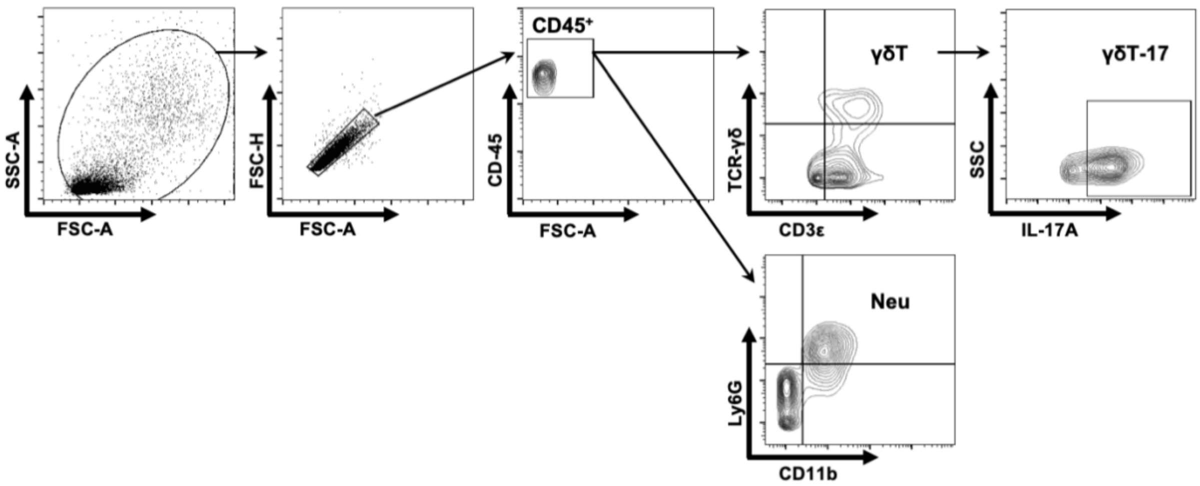

Supplementary Figure 2. Gating Strategy of flow cytometry assays. γδ T cells and neutrophils (Neu) were gated as CD3ε$^+$TCRγδ$^+$ and CD11b$^+$Ly6G$^+$, respectively, in CD45$^+$ BALF cells. γδ T-17 cells were IL-17A$^+$ subsets in CD3ε$^+$TCRγδ$^+$.

Supplementary Table 1. JHD was detected by HPLC with an optimal condition for obtaining maximum peaks. Relative retention time (RRT) and relative peak area (RPA) of each peak in the fingerprint of JHD extract.

No.	RRT	RPA (MV/s)	Identified compounds
**1**	3.831	39087210	
**2**	6.688	27296648	
**3**	12.973	38844341	Coffeic acid
**4**	21.170	46732876	Chlorogenic acid
**5**	23.044	58994287	

**6**	26.955	247089163	
**7**	28.763	28346592	
**8**	28.996	33474356	
**9**	31.982	22792791	
**10**	39.709	71133843	Rhein
**11**	50.798	39118417	Emodin

Supplementary Table 2. List of primers applied.

gene	Primer (5’-3’)
Il6-F	CCTACCCCAATTTCCAATGCTC
Il6-R	GGTCTTGGTCCTTAGCCACTC
Il1β-F	TGCCACCTTTTGACAGTGATG
Il1β-R	TGATGTGCTGCTGCGAGATT
Il17a-F	CCACGTCACCCTGGACTCTC
Il17a-R	CTCCGCATTGACACAGCG
Tnf-F	GTAGCCCACGTCGTAGCAAA
Tnf-R	ACAAGGTACAACCCATCGGC
Actb-F	CTCATGAAGATCCTGACCGAG
Actb-R	AGTCTAGAGCAACATAGCACAG
E. coli-F	GTTAATACCTTTGCTCATTGA

E. coli-R	ACCAGGGTATCTAATCCTGTT
Universal bacteria-F	ACGTCRTCCMCNCCTTCCTC
Universal bacteria-R	GTGSTGCAYGGYYGTCGTCA

Supplementary Table 3. Lung injury scoring system

Parameter	Score per field		
	0	1	2
A. Neutrophils in alveolar space	None	1-5	>5
B. Neutrophils in the interstitial space	None	1-5	>5
C. Hyaline membranes	None	1	>1
D. Proteinaceous debris filling the airspaces	None	1	>1
E. Alveolar septal thickening	<2x	2x-4x	>4x

Supplementary Table 4. Colon injury scoring system

Parameter	Score
Normal	0
Loss of goblet cell	1
Loss of goblet cell in large area	2
Loss of crypts	3
Loss of crypts in large area	4
